# Outcomes of Acute Kidney Injury among Hospitalized Patients with Infective Endocarditis: A National Inpatient Sample Analysis

**DOI:** 10.3390/jcm13144262

**Published:** 2024-07-22

**Authors:** Deepak Chandramohan, Boney Lapsiwala, Prathap Kumar Simhadri, Devansh Patel, Prabhat Singh, Sreekant Avula, Nihar Jena, Divya Chandramohan

**Affiliations:** 1 Department of Internal Medicine/Nephrology, University of Alabama at Birmingham, Paula Building, Room 235, 728 Richard Arrington Blvd S, Birmingham, AL 35233, USA; 2 Department of Medicine, Government Medical College, Surat 395001, India; boney0104@gmail.com; 3 Department of Nephrology, Advent Health, FSU College of Medicine, Daytona Beach, FL 32117, USA; prathap.simhadri@gmail.com; 4 Department of Internal Medicine/Nephrology, University of Alabama at Birmingham, Birmingham, AL 35294, USA; devanshhimanshupatel@uabmc.edu; 5 Department of Nephrology, Kidney Specialists of South Texas, Corpus Christi, TX 78404, USA; drprabhatsingh@hotmail.com; 6 Department of Internal Medicine/Endocrinology, University of Minnesota Twin Cities, Minneapolis, MN 55455, USA; drsreekanthavula@gmail.com; 7 Department of Internal Medicine/Cardiovascular Medicine, Trinity Health Oakland, Wayne State University, Pontiac, MI 48341, USA; niharjmd@gmail.com; 8 Department of Internal Medicine/Infectious Diseases, University of Texas Health San Antonio, San Antonio, TX 78229, USA; chandramohan@uthscsa.edu

**Keywords:** acute kidney injury, infective endocarditis, mortality, sepsis, hospital outcomes

## Abstract

**Background/Objectives**: Patients with infective endocarditis (IE) are more susceptible to acute kidney injury (AKI). The presence of AKI increases in-hospital complications in these patients. **Methods**: The 2016–2020 National Inpatient Sample (NIS) database consisting of adult admissions with IE and AKI was utilized. The primary outcome was all-cause inpatient mortality. Secondary outcomes included fluid and electrolyte disorders, stroke, septic arterial embolism, septic shock, cardiogenic shock, valve surgery, vasopressor support, mechanical ventilation, length of stay (LOS), and total hospital charges. **Results**: Out of a total of 63,725 adult admissions with IE, 16,295 (25.5%) admissions had AKI. Patients with AKI were more likely to be males (63% vs. 57.6%, *p* < 0.001) and older (55.8 vs. 50.4, *p* < 0.001). A higher proportion of these patients were admitted to large hospitals (60.6 vs. 55.3%, *p* < 0.001) and urban teaching hospitals (81.9 vs. 75%, *p* < 0.001). Patients with AKI had higher LOS (17 ± 16.1 vs. 11.32 ± 11.7, *p* < 0.001) and hospital charges (USD 239,046.8 ± 303,977.3 vs. USD 124,857.6 ± 192,883.5, *p* < 0.001). Multivariable analysis showed higher odds of all-cause inpatient mortality (aOR: 2.22, 95% CI: 1.81–2.73, *p* < 0.001). They also had higher risk for fluid and electrolyte disorder (aOR: 2.31, 95% CI: 2.10–2.53, *p* < 0.001), septic arterial embolism (aOR: 1.61, 95% CI: 1.42–1.84, *p* < 0.001), septic shock (aOR: 3.78, 95% CI: 2.97–4.82, *p* < 0.001), cardiogenic shock (OR: 3.37, 95% CI: 2.65–4.28, *p* < 0.001), valve surgery (aOR: 1.52, 95% CI: 1.35–1.71, *p* < 0.001), vasopressor requirement (aOR: 1.99, 95% CI: 1.52–2.60, *p* < 0.001), and mechanical ventilation (aOR: 2.75, 95% CI: 2.33–3.24, *p* < 0.001). The association with stroke was elevated but not statistically significant. **Conclusions**: This large retrospective analysis demonstrated that patients with AKI and infective endocarditis had increased mortality, adverse hospital outcomes, increased LOS, and hospital costs.

## 1. Introduction

Infective endocarditis (IE) is a potentially fatal infection associated with high mortality [1]. Its global prevalence has more than doubled between 1990 and 2019 despite advances in diagnosis and treatments, and the mortality is close to 25% [2]. There has also been an increase in the morbidity related to the disease, as well as in healthcare costs [3,4].

Acute kidney injury (AKI) is a well-known complication of IE [5]. The common etiologies of acute kidney injury (AKI) in infective endocarditis (IE) can be classified into three main categories: (a) infection-related causes (such as sepsis, embolism leading to renal infarcts, and occasionally glomerulonephritis), (b) diagnostics-related causes (resulting from the use of intravenous contrast agents), and (c) treatment-related causes (including the use of antibiotics, analgesics, and diuretics) [6]. AKI due to infection-related etiologies occurs more commonly than other causes [5]. Multiple studies have demonstrated that AKI is linked to negative consequences in both the short and long term. This effect is seen even in patients who develop non-severe AKI [7]. The pathogenesis of septic AKI is intricate and encompasses inflammation, oxidative stress, and microvascular dysfunction with further exacerbation of injury through the production of cytokines by tubular cells. Recent findings indicate that AKI serves as a marker for sickness severity and a catalyst for multi-organ failure [8]. The definition of AKI has been updated over the years. The commonly used definitions include Kidney Disease Improving Global Outcomes (KDIGO), Risk, Injury, Failure, Loss of Kidney Function, and End-stage Kidney Disease (RIFLE), and the Acute Kidney Injury Network (AKIN) [9].

AKI requiring dialysis (AKI-D) is a severe form of AKI associated with long-term ramifications. Patients who need dialysis due to AKI are thrice as likely to require chronic dialysis [10]. For these reasons, the detection of AKI and knowledge about outcomes would help in risk stratification. This study aimed to assess the outcomes of patients with IE and AKI. We also evaluated the factors responsible for AKI requiring dialysis using a large database. 

## 2. Materials and Methods

### 2.1. Data Source

**Figure 1:**
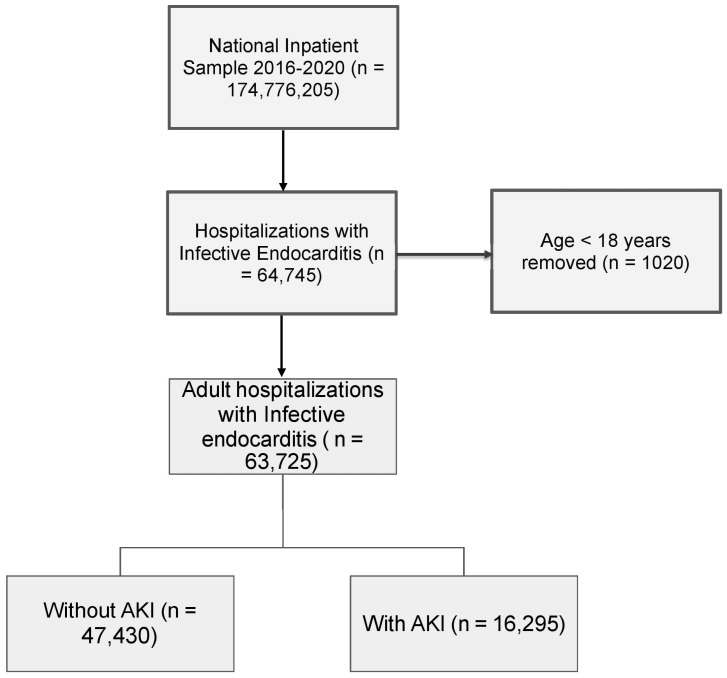
Patient selection (weighted) flowchart. Abbreviations: AKI—acute kidney injury.

We utilized the National Inpatient Sample (NIS) databases during the years 2016–2020 of the Healthcare Cost and Utilization Project (HCUP) sponsored by the Agency for Healthcare Research and Quality (AHRQ) [11]. The NIS is a large publicly available dataset in the United States that contains data representative of approximately 20% of the inpatient healthcare services provided to patients admitted to hospitals from over 48 states. We used International Classification of Diseases Clinical Modification codes (ICD-10-CM) to identify patients ≥ 18 years old with a primary diagnosis of IE and a secondary diagnosis of AKI [12]. The ICD-10-CM codes used are included in the Table S1 in supplementary information. Groups were created based on the presence or absence of AKI. The Elixhauser comorbidity index was used to compare the comorbidities between groups [13]. The patient selection process is shown in Figure 1. Due to deidentified data in the NIS, obtaining approval from the Institutional Review Board (IRB) was not required. 

### 2.2. Outcomes Assessed

The primary outcome assessed was all-cause inpatient mortality. The secondary outcomes included fluid and electrolyte disorders, stroke, septic arterial embolism, septic shock, cardiogenic shock, valve surgery, vasopressor support, mechanical ventilation, cardiac arrest, length of stay (LOS), and total hospital charges due to AKI in IE. 

### 2.3. Statistical Analysis

Continuous variables were represented by their means and standard deviations. Categorical variables were represented by their frequencies and percentages. The baseline characteristics were compared using a *t*-test for continuous variables and the chi-square test for categorical variables. A *p*-value less than 0.05 was considered statistically significant. A univariable regression analysis calculated the crude odds ratio for factors linked to AKI in the IE cohort. Subsequently, multivariable logistic regression models were created to compute the adjusted odds ratio (aOR). The models were built by incorporating the variables identified as predictors of association in univariable analysis and from variables previously found to have an association according to the existing literature [5,14]. The statistical analysis was conducted using Stata/MP, version 18 (StataCorp, College Station, TX, USA) [15]. 

## 3. Results

### 3.1. Patient Characteristics

A total of 63,725 admissions for infective endocarditis were identified out of 174,776,205 admissions during 2016–2020 by applying the inclusion and exclusion criteria. A total of 16,295 (25.5%) had AKI out of these 63,725 admissions. The mean age of patients with AKI was higher (55.8 vs. 50.4 years), and males were more commonly affected. Medicare beneficiaries (41.2 vs. 33.7%, *p* < 0.001) and uninsured patients (6.9 vs. 3.6%, *p* < 0.001) more commonly had AKI. The two groups had no significant differences in race, median household for patient’s zip code, and hospital region. Compared to patients without AKI, patients with AKI had more admissions to large hospitals (60.6% vs. 55.3) and urban teaching hospitals (81.9 vs. 75%). Among comorbidities, patients with AKI had increased rates of diabetes mellitus (DM) with complications (18.1 vs. 9.9%), congestive heart failure (CHF) (50.5% vs. 28.1%), cardiac arrhythmias (45.5 vs. 31.6%), cerebrovascular disease (20% vs. 13.7%), chronic kidney disease (CKD) (36.2 vs. 13.6%), liver disease (3.3 vs. 1.4%), and obesity (15 vs. 10.3%). The Elixhauser comorbidity index *score of ≥5 was* significantly higher in patients with AKI (73 vs. 44%). The AKI group had fewer patients with arterial hypertension (AH) and opioid use than the non-AKI group. The baseline characteristics are shown in Table 1. and the factors associated with AKI are shown in Table 2. 

### 3.2. Factors Associated with AKI-D

Additional analysis was performed to determine the factors associated with AKI-D. The number of patients who required dialysis was 1425 (8.7%). There was an association with Blacks (aOR: 1.61, 95% CI: 1.06–2.44, *p* = 0.024), median-income populations, the 25th to 50th quartile and the 50th to 75th quartile compared to the 0 to 25th quartile, and medium and large hospitals when compared to smaller hospitals. Patients with DM with complications (aOR: 1.65, 95% CI: 1.20–2.28, *p* = 0.002), Elixhauser comorbidity index score of ≥5 (aOR: 2.65, 95% CI: 1.75–4.02, *p* < 0.001), and prior prosthetic valve (aOR: 2.79, 95% CI: 1.78–4.37, *p* < 0.001) were associated with AKI-D. Compared to CKD stage 2 patients, CKD stage 5 patients had a higher risk (aOR: 14.7, 95% CI: 2.46–88.59, *p* = 0.003). CKD stages 3 and 4 patients were also at high risk (aOR: 2.19 and 2.94), but the results were not statistically significant. *S. aureus* and gram-negative infections were also associated with AKI-D but were not statistically significant. The results are summarized in Table 3. 

### 3.3. Outcomes

**Figure 2:**
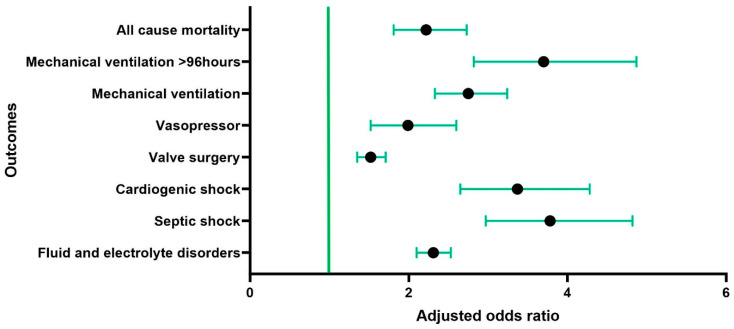
Forest plot showing in-hospital outcomes of patients with acute kidney injury (AKI) in infective endocarditis (IE).

Patients with AKI had high all-cause inpatient mortality (aOR: 2.22, 95% CI: 1.81–2.73, *p* < 0.001); they were more likely to experience fluid and electrolyte disorders (aOR: 2.31, 95% CI: 2.10–2.53, *p* < 0.001), septic arterial embolism (aOR: 1.61, 95% CI: 1.42–1.84, *p* < 0.001), septic shock (aOR: 3.78, 95% CI: 2.97–4.82, *p* < 0.001), cardiogenic shock (OR: 3.37, 95% CI: 2.65–4.28, *p* < 0.001), valve surgery (aOR: 1.52, 95% CI: 1.35–1.71, *p* < 0.001), vasopressor requirement (aOR: 1.99, 95% CI: 1.52–2.60, *p* < 0.001), mechanical ventilation (aOR: 2.75, 95% CI: 2.33–3.24, *p* < 0.001), and mechanical ventilation >96 h (aOR: 3.7, 95% CI: 2.82–4.87, *p* < 0.001). Patients with AKI had an increased association with stroke, but it was not statistically significant. There was an increased LOS of 17 ± 16.11 days and associated total hospital charges of USD 23,9046.8 ± 303,977.3. The results of the multivariable analysis are summarized in Table 4 and Figure 2. 

## 4. Discussion

In our population-based study using data from the NIS, we assessed the associations and outcomes among patients with AKI in IE. The significantly increased risk of all-cause inpatient mortality and other complications in patients with AKI compared to patients without AKI shows that AKI substantially elevates the risk of poor outcomes in these patients. In addition, we also found several associations with AKI. Older adults, urban teaching, large hospitals, history of congestive heart failure, complicated DM, CHF, cardiac arrhythmias, cerebrovascular disease, CKD, moderate to severe liver disease, and Elixhauser comorbidity index score of >5 were all associated with AKI in this population. The Elixhauser comorbidity index predicts in-hospital mortality and the risk of readmission [13].

A French study by Gagneux-Brunon et al., using the KDIGO criteria to define AKI, noted that the incidence of AKI in their cohort of IE was 68.7%, and their mortality rate was around 10% [5]. Our study demonstrated a much lesser incidence of 25.5% but a similar mortality rate of 8.7%. In another study by Tokarski et al., vasopressor use was associated with AKI, especially early AKI [14]. Our analysis also demonstrated that these patients had a high rate of intensive care unit (ICU) admissions requiring vasopressors and mechanical ventilation. Moreover, they needed a longer duration of mechanical ventilation > 96 h.

AKI due to hemodynamic disturbances is common in IE. These hemodynamic disturbances may arise either from the systemic effect of the infection or from the cardiogenic complications associated with IE, most notably congestive heart failure, which may lead to reduced renal blood flow and subsequent renal injury [14,16]. Studies performed on animal models have demonstrated that AKI can further cause tissue damage to the heart [7]. Cardiac complications, such as valvular dysfunction and heart failure due to IE, could result in reduced cardiac output, which in turn reduces the renal perfusion pressure, resulting in prerenal azotemia and a continuum of AKI [17]. Heart failure is seen in about 42–60% of the cases, and moderate to severe heart failure is a predictor of mortality within the first year [18]. We found that there was a high risk for cardiogenic shock in these patients. 

In our study, we found that women were less likely to develop AKI due to IE; this was also seen by Torkarski et al. [14]. Among different age groups, patients ≥ 60 had a higher rate of AKI. However, age was not associated with AKI in a study by Legrand et al. [19]. Interestingly, we observed significant regional differences in hospitalization rates, which had only been previously described by Bor et al. Prior studies evaluating AKI in IE have not reported such discrepancies. While the exact reason is unknown, this could likely be due to the varied incidence of IE in previous studies [20]. 

Risk factors for AKI such as age, hypertension, DM, thrombocytopenia, S. aureus infections, peripheral arterial disease, cardiac failure, nor-epinephrine use, and vancomycin exposure have been described in prior studies [5,14,21,22]. Our study did not find an increased association with AH or peripheral arterial disease, but there were increased associations with DM, CHF, cerebrovascular disease, CKD, and liver disease. In our study, a high Elixhauser comorbidity index score was also associated with AKI, similar to Ortiz-Soriano et al. [23]. 

Another significant contributing factor to the occurrence of AKI is the risk for nephrotoxicity due to antibiotics such as vancomycin, especially when co-administered with tazobactam/piperacillin [24]. Although nephrotoxicity is common with aminoglycosides, concomitant administration with vancomycin causes a similar increase in the risk of AKI and is therefore not recommended [22,25]. Beta-lactams and fluoroquinolones can cause AKI due to acute interstitial nephritis [25]. 

Pre-existing kidney disease complicates infective endocarditis (IE) in patients and is an important risk factor for AKI. The mechanisms leading to this higher vulnerability are reduced renal reserve and altered hemodynamic responses. Furthermore, antibiotics such as aminoglycosides could cause an increased nephrotoxic insult in patients in the presence of CKD [14,26]. 

*S. aureus* is the organism responsible for most cases of IE. Staphylococcus, through superantigens, can incite an immune response, causing AKI [27]. Unlike a previous study by Legrand et al. that showed no association of post-operative AKI in patients with infective endocarditis due to S. aureus, our study found an association between the two [19]. *S. aureus* has also been linked to increased neurological complications such as stroke and brain abscess. Neurological complications warrant early surgery and other interventions [18]. 

Our study also demonstrated some key associations with AKI-D. There were certain demographic associations among patients who developed AKI-D, such as Black race, regions other than the northeast, medium and large hospitals, and urban hospitals. Increased rates of CKD, hypertension, diabetes mellitus, and poor access to healthcare could be the reason for Blacks having increased risk for AKI-D. Among comorbidities, there were associations with complicated diabetes and CKD stage 5. CKD has been implicated as a significant risk factor for AKI [9,16]. Early onset of AKI is a risk factor for increased mortality in the first year and progression to CKD [14]. Prior prosthetic valve was associated with increased AKI-D in our study, similar to Gagneux-Brunon et al. [5]. A study by Petersen et al. showed that the mortality rate among hospitalized patients in Denmark with AKI -D was 40.4%. It was also noted that about 21.6% required dialysis continuation even after discharge [26]. The rate of AKI-D was reported to have risen from 3.1% to 4.2% between 2003 and 2016. Our study showed a much higher rate, around 8.7%, during the years from 2016 to 2020 [3]. 

IE admissions have been associated with increased costs in recent years. The overall increase in the healthcare expenditure towards IE hospitalizations is mainly due to an increase in the hospitalizations of these patients [3]. Another NIS study by Silver et al. evaluated all AKI hospitalizations due to various etiologies and found that the hospitalization costs were not only higher in AKI, but higher compared to patients with myocardial infarction or gastrointestinal bleeding [28]. This was also shown in the study by Ortiz-Soriano et al., who found that hospital costs increased based on the severity of AKI in patients with IE. There was an increase in cost by 65.3% in patients when stage 1 AKI was compared to stage ≥ 2 AKI [23]. Our study had similar findings and found that patients with AKI have more healthcare expenditures.

## 5. Strengths and Limitations

Our study has some limitations. The NIS database provides longitudinal data, but the retrospective study design has its limitations. First, since retrospective analysis relies on the accuracy of the data in the dataset, there exists a possibility for some inaccuracies. Second, misclassification bias and selection bias could be present in the dataset. Third, the diagnosis of AKI could have been made based on different criteria by clinicians. There is also a possibility that subclinical AKI could not have been documented. Finally, the validation of microbiology data is inadequate in retrospective studies based on NIS (3). Nonetheless, our study also has its merits; it was conducted on a large population without any referral bias, and numerous potential confounders were accounted for in the multivariable analysis. 

## 6. Conclusions

To our knowledge, this is the first study looking at the outcomes of AKI in hospitalized patients in the United States using a large database. In patients with IE, the prevalence of AKI was present in 25.5%, and of these, 8.7% required dialysis. AKI in IE was associated with increased all-cause mortality and other complications. There was an associated increase in hospital LOS and hospital charges. Large randomized controlled trials are needed to investigate potential risk factors for AKI and dialysis in this population. Patients who develop AKI should be monitored closely for other complications. Early detection, risk stratification, and interventions could help decrease mortality and morbidity in IE. 

## Figures and Tables

**Table 1:** Weighted patient characteristics and comorbidities of adult hospitalizations with infective endocarditis (IE) and acute kidney injury (AKI) from the NIS between 2016 and 2020.

Characteristics		Without AKI (n = 47,430)	With AKI (n = 16,295 (25.5%))	*p*-Value
Age in years, Mean ± SD		50.46 ± 19.08	55.81 ± 18.48	<0.001
Age group				<0.001
	18–44 yrs	20,805 (43.9)	5195 (31.8)	
	45–59 yrs	10,165 (21.4)	3645 (22.3)	
	60–74 yrs	10,065 (21.2)	4455 (27.3)	
	75 yrs or older	6395 (13.5)	3000 (18.4)	
Gender				<0.001
	Female	20,075 (42.4)	6040 (37)	
	Male	27,340 (57.6)	10,255 (63)	
Race				0.0875
	White	35,340 (74.5)	12,100 (74.2)	
	African American	4810 (10.1)	1610 (9.8)	
	Hispanic	3630 (7.6)	1200 (7.3)	
Median household income for patient’s zip code				0.009
	Quartile 1	15,330 (32.3)	5165 (32.5)	
	Quartile 2	12,465 (26.3)	3895 (24.5)	
	Quartile 3	10,175 (21.5)	3845 (24.4)	
	Quartile 4	8245 (17.4)	2960 (18.6)	
Hospital region				0.164
	Northeast	10,700 (22.6)	3510 (21.5)	
	Midwest	9770 (20.6)	3650 (22.3)	
	South	18,365 (38.7)	6295 (38.6)	
	West	8595 (18.1)	2840 (17.4)	
Hospital size				<0.001
	Small	8620 (18.1)	2360 (14.4)	
	Medium	12,600 (26.6)	4045 (24.8)	
	Large	26,210 (55.3)	9890 (60.6)	
Hospital location and teaching status				<0.001
	Rural	3385 (7.1)	725 (4.4)	
	Urban non-teaching	8460 (17.9)	2220 (13.6)	
	Urban teaching	35,585 (75)	13,350 (81.9)	
Medical Insurance				<0.001
	Medicare	15,985 (33.7)	6715 (41.2)	
	Medicaid	15,960 (33.6)	4735 (29)	
	Private	9750 (20.5)	3140 (19.2)	
	No insurance	1715 (3.6)	1130 (6.9)	
Elixhauser comorbidity Index				<0.001
	0	635 (1.3)	0	
	1	3225 (6.8)	163 (1)	
	2	6155 (13)	652 (4)	
	3–4	16,515 (34.9)	3585 (22)	
	>5	20,900 (44)	11,896 (73)	
Disposition				0.997
	Discharge home	13,020 (27.4)	2933 (18)	
	Discharge to SNF, ICF, LTACH	11,395 (24)	5703 (35)	
	Discharge to HHC	9930 (20.9)	3096 (19)	
Microorganism				
	Staphylococcus	15,185 (32)	5315 (32.6)	0.524
	Streptococcus	9430 (19.9)	2495 (15.3)	<0.001
	Enterococcus	5170 (10.8)	2120 (13)	0.001
	Gram-negative	5210 (10.9)	2110 (12.9)	0.002
Comorbidities				
	Arterial hypertension	15,710 (33.1)	4700 (28.8)	<0.001
	Diabetes mellitus	4875 (10.2)	1680 (10.3)	0.958
	Diabetes mellitus with complications	4720 (9.9)	2950 (18.1)	<0.001
	Hyperlipidemia	11,730 (24.7)	4429 (27.1)	0.006
	Congestive heart failure	13,360 (28.1)	8240 (50.5)	<0.001
	Peripheral vascular disease	3580 (7.5)	1470 (9)	0.007
	Coronary artery disease	9005 (18.9)	4185 (25.6)	<0.001
	Cardiac arrhythmias	14,995 (31.6)	7425 (45.5)	<0.001
	Cerebrovascular disease	6545 (13.7)	3265 (20)	<0.001
	Chronic kidney disease	6455 (13.6)	5900 (36.2)	<0.001
	Fluid and electrolyte disorders	15,620 (32.9)	9840 (60.3)	<0.001
	Chronic obstructive pulmonary disease	8365 (17.6)	3325 (20.4)	<0.001
	Moderate/severe liver disease	685 (1.4)	550 (3.3)	<0.001
	Rheumatological disorders	1260 (2.6)	505 (3)	0.193
	Anemia	5145 (10.8)	2130 (13)	<0.001
	Dementia	1340 (2.8)	615 (3.7)	0.006
	Cancer	1165 (2.4)	575 (3.5)	0.001
	Metastatic cancer	685 (1.4)	235 (1.4)	0.139
	Obesity	4905 (10.3)	2460 (15)	<0.001
	Smoking	17,355 (36.5)	4364 (26.7)	<0.001
	Opioid use	13,320 (28)	3485 (21.3)	<0.001
	Prosthetic valve	1840 (3.8)	810 (4.9)	0.008
Resource utilization				
	LOS, mean ± SD	11.32 ± 11.74	17 ± 16.11	<0.001
	Total charges, mean ± SD	124,857.6 ± 192,883.5	239,046.8 ± 303,977.3	<0.001

Abbreviations: HHC, home with health care; ICF, intermediate care facility; LTACH, long-term acute care hospital; SD, standard deviation; SNF, skilled nursing facility.

**Table 2:** Factors associated with AKI in infective endocarditis.

Variable		Multivariable Adjusted OR (95% CI)	*p*-Value
Age		1.01 (1.0–1.01)	<0.001
Sex	Female	0.79 (0.72–0.87)	<0.001
Race (reference Caucasian)			
	Black	0.77 (0.67–0.90)	0.001
	Hispanic	0.88 (0.74–1.05)	0.161
Median household income for patient’s zip code % (reference quartile 1)			
	Quartile 2	0.93 (0.83–1.05)	0.297
	Quartile 3	1.1 (0.97–1.24)	0.134
	Quartile 4	1.02 (0.89–1.18)	0.686
Elixhauser comorbidity index (reference 0)			
	1	1.26 (0.42–3.73)	0.669
	2	2.63 (0.94–7.36)	0.065
	3–4	5.89 (2.13–16.22)	0.001
	≥5	14.37 (5.22–39.55)	<0.001
Region (reference northeast)			
	Midwest	0.99 (0.86–1.14)	0.940
	South	1.08 (0.95–1.23)	0.206
	West	0.93 (0.80–1.08)	0.373
Hospital bed size (reference small)			
	Medium hospital	1.12 (0.97–1.29)	0.099
	Large hospital	1.26 (1.11–1.43)	<0.001
Hospital location and teaching status (reference rural hospital non-academic)			
	Urban non-teaching	1.13 (0.89–1.42)	0.292
	Urban teaching	1.47 (1.19–1.82)	<0.001
Insurance (reference Medicare)			
	Medicaid	1.31 (1.13–1.51)	<0.001
	Private	1.15 (1.01–1.31)	0.035
	No insurance	1.36 (1.10–1.67)	0.004
Organism			
	Staphylococcus	1.25 (1.13–1.38)	<0.001
	Streptococcus	0.70 (0.62–0.79)	<0.001
	Enterococcus	0.92 (0.80–1.05)	0.236
	Gram-negative	1.12 (0.98–1.28)	0.082
Comorbidities			
	Arterial hypertension	0.55 (0.50–0.61)	<0.001
	Diabetes mellitus	0.71 (0.62–0.82)	<0.001
	Diabetes mellitus with complications	1.31 (1.15–1.49)	<0.001
	Hyperlipidemia	0.72 (0.64–0.80)	<0.001
	Congestive heart failure	1.58 (1.43–1.75)	<0.001
	Peripheral vascular disease	0.78 (0.67–0.91)	0.002
	Coronary artery disease	0.93 (0.83–1.04)	0.235
	Cardiac arrhythmias	1.06 (0.97–1.17)	0.164
	Cerebrovascular disease	1.21 (1.08–1.36)	0.001
	Chronic kidney disease	2.6 (2.41–3.01)	<0.001
	Chronic obstructive pulmonary disease	0.84 (0.75–0.94)	0.003
	Moderate/severe liver disease	1.52 (1.16–2.0)	0.002
	Rheumatological disorders	0.91 (0.70–1.17)	0.483
	Anemia	0.98 (0.86–1.12)	0.986
	Dementia	0.97 (0.76–1.23)	0.827
	Cancer	1.03 (0.81–1.32)	0.778
	Metastatic cancer	0.79 (0.55–1.15)	0.23
	Obesity	1.08 (0.95–1.23)	0.192
	Smoker	0.73 (0.65–0.82)	<0.001
	Opioid use	0.84 (0.74–0.96)	0.015
	Prosthetic valve	0.98 (0.79–1.22)	0.903

Multivariable analysis was performed by adjusting for age, sex, race, median income for zip code, Elixhauser comorbidity index, hospital bed size, hospital location, teaching status, and insurance.

**Table 3:** Factors associated with acute kidney injury requiring hemodialysis in infective endocarditis, n = 1425.

Variable		Multivariable Adjusted OR (95% CI)	*p*-Value
Age		0.98 (0.97–0.99)	*0.019*
Female		0.81 (0.611.08)	*0.160*
Race (reference Caucasian)			
	Black	1.61 (1.06–2.44)	*0.024*
	Hispanic	1.28 (0.77–2.13)	*0.325*
Median household income for patient’s zipcode % (reference quartile 1)			
	Quartile 2	1.41 (0.98–2.03)	*0.059*
	Quartile 3	1.50 (1.02–2.20)	*0.036*
	Quartile 4	1.37 (0.90–2.07)	*0.137*
Elixhauser comorbidity index (reference 0)			
	≥5	2.65 (1.75–4.02)	*<0.001*
Region (reference Northeast)			
	Midwest	2.03 (1.32–3.11)	*0.001*
	South	1.75 (1.17–2.61)	*0.006*
	West	1.29 (0.80–2.10)	*0.291*
Hospital bedsize (reference small)			
	Medium hospital	1.93 (1.61–3.23)	*0.011*
	Large hospital	1.91 (1.19–3.04)	*0.007*
Hospital location and teaching status (reference rural hospital non academic)			
	Urban non-teaching	1.96 (0.74–5.15)	*0.171*
	Urban teaching	1.91 (0.77–4.73)	*0.162*
Insurance (reference Medicare)			
	Medicaid	0.73 (0.47–1.13)	*0.168*
	Private	0.67 (0.43–1.05)	*0.083*
	No insurance	0.43 (0.22–0.86)	*0.018*
Prosthetic valve		2.79 (1.78–4.37)	<0.001
Organism			
	Staphylococcus	1.13 (0.85–1.50)	0.390
	Streptococcus	0.71 (0.48–1.05)	0.094
	Enterococcus	0.89 (0.60–1.32)	0.565
	Gram neg	1.15 (0.78–1.68)	0.460
Chronic kidney disease stage (reference stage 2)			
	Chronic kidney disease stage 3	2.19 (0.48–9.92)	0.309
	Chronic kidney disease stage 4	2.94 (0.60–14.42)	0.183
	Chronic kidney disease stage 5	14.76 (2.46–88.59)	0.003
Other Comorbidities			
	Arterial hypertension	0.50 (0.35–0.72)	*<0.001*
	Diabetes mellitus	0.59 (0.36–0.97)	*0.038*
	Diabetes mellitus with complications	1.65 (1.20–2.28)	*0.002*
	Hyperlipidemia	0.98 (0.70–1.37)	*0.933*
	Congestive heart failure	1.19 (0.89–1.59)	0.217
	Peripheral vascular disease	0.87 (0.55–1.38)	0.582
	Coronary artery disease	0.86 (0.61–1.21)	0.408
	Cardiac arrhythmias	1.17 (0.89–1.54)	0.253
	Cerebrovascular disease	1.31 (0.96–1.79)	0.086
	Fluid and electrolyte disorders	2.09 (1.53–2.85)	<0.001
	Chronic obstructive pulmonary disease	0.75 (0.53–1.05)	0.103
	Moderate/severe liver disease	0.72 (0.32–1.59)	0.422
	Rheumatological disorders	0.83 (0.39–1.77)	0.639
	Anemia	0.66 (0.43–1.02)	0.065
	Dementia	0.34 (0.12–0.98)	0.046
	Cancer	0.86 (0.42–1.75)	0.694
	Metastatic cancer	0.43 (0.09–1.92)	0.271
	Obesity	1.25 (0.90–1.75)	0.171
	Smoker	0.73 (0.50–1.05)	0.092

Multivariable analysis was performed by adjusting for age, sex, race, median income for zip code, Elixhauser comorbidity index, hospital bed size, hospital location, teaching status and insurance.

**Table 4:** Outcomes of infective endocarditis (IE) with acute kidney injury (AKI).

Outcomes	AKI with IE, N(%)	Multivariable Adjusted OR (95% CI)	*p*-Value
Fluid and electrolyte disorders	9840 (60.3)	2.31 (2.10–2.53)	<0.001
Stroke	370 (2.2)	1.10 (0.76–1.57)	0.597
Septic arterial embolism	3150 (19.3)	1.61 (1.42–1.84)	<0.001
Septic shock	1375 (8.4)	3.78 (2.97–4.82)	<0.001
Cardiogenic shock	1385 (8.5)	3.37 (2.65–4.28)	<0.001
Valve surgery	4900 (30)	1.52 (1.35–1.71)	<0.001
Vasopressor requirement	800 (4.9)	1.99 (1.52–2.60)	<0.001
Mechanical ventilation	2580 (15.8)	2.75 (2.33–3.24)	<0.001
Mechanical ventilation > 96 h	1130 (6.9)	3.7 (2.82–4.87)	<0.001
Mortality	1430 (8.7)	2.22 (1.81–2.73)	<0.001

Multivariable analysis was performed by adjusting for age, sex, race, hospital bed size, hospital location and teaching status, insurance, hypertension, diabetes, hyperlipidemia, peripheral vascular disease, cerebrovascular disease, chronic kidney disease, fluid and electrolyte disorders, chronic obstructive pulmonary disease, moderate–severe liver disease, and smoking.

## Data Availability

The National Inpatient Sample data are publicly available at the HCUP-US Home Page (ahrq.gov).

## Supplementary Materials

The following supporting information can be downloaded at https://www.mdpi.com/article/10.3390/jcm13144262/s1, Table S1: List of ICD-10 codes utilized in the present study.
