# Supplementary material for: Outcomes of Acute Kidney Injury among Hospitalized Patients with Infective Endocarditis: A National Inpatient Sample Analysis
Source: J Clin Med. 2024 Jul 22;13(14):4262. doi: 10.3390/jcm13144262 (PMC11278004; doi:10.3390/jcm13144262)
Supplement: Supplementary file 1 [file jcm-13-04262-s001.zip › jcm-3074282-supplementary.pdf]

Supplementary Materials:

Table S1: List of ICD-10 codes utilized in the present study

Diagnosis/Procedure	ICD 10 codes
Infective endocarditis	I33
Acute kidney injury	N17
Hypertension	H35031, H35032, H35033, H35039, I10, I110, I119, I674, O111, O112, O113, O119, O114, O115, O1001, O1002, O1003, O10111, O10112, O10113, O10119, O161, O162, O163, O164, O165, O169, I160, I161, I169
Diabetes mellitus	E10.0, E10.1, E10.6, E10.8, E10.9, E11.0, E11.1, E11.6, E11.8, E11.9, E12.0, E12.1, E12.6, E12.8, E12.9, E13.0, E13.1, E13.6, E13.8, E13.9, E14.0, E14.1, E14.6, E14.8, E14.9
Diabetes mellitus with complications	E10.2–E10.5, E10.7, E11.2–E11.5, E11.7, E12.2–E12.5, E12.7, E13.2 E13.5, E13.7, E14.2–E14.5, E14.7
Hyperlipidemia	E78
Congestive heart failure	I09.9, I11.0, I13.0, I13.2, I25.5, I42.0, I42.5–I42.9, I43.x, I50.x, P29.0
Peripheral vascular disease	I70.x, I71.x, I73.1, I73.8, I73.9, I77.1, I79.0, I79.2, K55.1, K55.8, K55.9, Z95.8, Z95.9
Coronary artery disease	I25, I254

Cardiac Arrhythmia	I44.1–I44.3, I45.6, I45.9, I47–I49, R00.0, R00.1, R00.8, T82.1, Z45.0, Z95.0
Cerebrovascular disease	I69, G454, G458, G459, I672, I679, I6782, I6789, I6781, Z8673
Chronic obstructive pulmonary disease	I27.8, I27.9, J40.x–J47.x, J60.x–J67.x, J68.4, J70.1, J70.3
Moderate/Severe liver disease	I85.0, I85.9, I86.4, I98.2, K70.4, K71.1, K72.1, K72.9, K76.5, K76.6, K76.7
Rheumatological disorders	M05.x, M06.x, M31.5, M32.x–M34.x, M35.1, M35.3, M36.0
Anemia	D51, D52, D53, D500, D508, D509
Cancer	C00.x–C26.x, C30.x–C34.x, C37.x C41.x, C43.x, C45.x–C58.x, C60.x C76.x, C81.x–C85.x, C88.x, C90.x–C97.x
Metastatic cancer	C77.x–C80.x
Obesity	E66, Z6830, Z6831, Z6832, Z6833, Z6834, Z6835, Z6836, Z6837, Z6838, Z6839, Z6841, Z6842, Z6843, Z6844, Z6845, Z684
Smoking	F17, Z720

Septic arterial embolism	I76
Mechanical ventilation	5A1935Z, 5A1945Z, 5A1955Z
Mechanical ventilation >96 hours	5A1955Z, Z9911
